# The role of artificial intelligence in thyroid cytology of indeterminate nodules: from digital cytology to multimodal precision triage

**DOI:** 10.3389/fendo.2026.1800918

**Published:** 2026-07-14

**Authors:** Pietro Tralongo, Mariagiovanna Ballato, Vincenzo Fiorentino, Valeria Zuccalà, Cristina Pizzimenti, Ludovica Rita Pepe, Angelica Cardile, Teresa Maria Martorana, Antonio Ieni, Maurizio Martini, Guido Fadda

**Affiliations:** 1 Department of Biomedical, Dental and Morphological and Functional Imaging Sciences, University of Messina, Messina, Italy; 2 Department of Human Pathology of the Adulthood and of the Developing Age “Gaetano Barresi”, University of Messina, Messina, Italy; 3 Pathology Unit, Papardo Hospital, Messina, Italy

**Keywords:** AI-assisted analysis, cytology, indeterminate nodules, thyroid, whole slide analysis

## Abstract

Indeterminate thyroid cytology is among the most challenging bottlenecks in thyroid nodule management and continues to be a significant source of risk stratification ambiguity and potentially preventable diagnostic surgeries. While molecular analysis has helped refine preoperative risk stratification, especially among Bethesda III (AUS) and Bethesda IV (FN/SFN) thyroid nodules, issues remain regarding positive predictive value, availability, and subsequent malignancy risk, even among those having the “negative” molecular risk assessment. The field of artificial intelligence (AI) is presently experiencing an accelerated trajectory of expansion into thyroid diagnostic fields, extending initially from thyroid ultrasound into the realms of whole-slide cytology analysis and computational pathology. The purpose of this narrative review is to highlight the changing landscape of AI applications in the context of the workup of indeterminate thyroid nodules, focusing on thyroid cytology and decision support for indeterminate thyroid nodules. It is becoming clear from the evidence that AI has the potential to decrease subjectivity and variability in cytology/Whole-Slide Imaging (WSI) analysis, optimize the selection of candidates for biopsy in the upstream process, and optimize post-FNA evaluation by fusion of molecular analysis in the downstream process. Future application will depend upon validation, standardization, and prospectively conducted studies in patients.

## 1 Why indeterminate thyroid cytology remains clinically challenging

Thyroid fine-needle aspiration (FNA) remains the mainstay of thyroid nodule diagnosis, while traditional cytologic analysis has inherent challenges in follicular-patterned lesions, in which architectural features necessary for the evaluation of malignancy are not adequately assessed in cytologic samples (1, 2). While there has been an improved integration of results in management, the term ‘indeterminate’ remains in the diagnostic gray zone fueling diverse practices and differences in surgery rates (3). Outside of interpretive ambiguity, indeterminate cytopathology also has system-wide consequences including increasing the rate of repeat FNA procedures, specialist consultations, and requests for molecular testing and lobectomies, among them a significant percentage being benign. The ‘gray-zone’ burden also becomes more significant given the influence of contemporary practice conditions that increasingly have small and incidentally detected nodules being sampled and detected, for whom the balance between risks and benefits of surgical procedures is critical. Thus, studies focusing on issues regarding higher reproducibility and triage, as against issues of maximizing ‘accuracy,’ are most relevant for any intervention by AI (4).

The Bethesda System for Reporting Thyroid Cytopathology (TBSRTC) offers an integral scheme correlating cytopathological diagnoses with an estimate of Risk of Malignancy (ROM), and to clinical management recommendations. The most recent version of the guidelines (TBSRTC 2023) not only further defines enhanced criteria, but the ROM of Bethesda III (AUS) and Bethesda IV (FN/SFN) still display broad intersite variability, because of subjective criteria and local variations of disease prevalence (5, 6). Previous meta-analyses implied, in fact, an underestimation of malignancy in indeterminate cytopathologic results in the various Bethesda classes, because ROM in these criteria evolves over time due to improvements in histological reclassifications (7).

Another critical influence is NIFTP, which altered the meaning of “malignancy” outcomes and added complexity to how cytology is linked to clinically meaningful endpoints, for instance, “requires surgery” versus “clinically relevant cancer” (8). In practice, several pitfalls exist that cytopathologists commonly face, such as borderline nuclear atypia, oncocytic change, thyroiditis-related atypia, cystic degeneration, and low cellularity, all of which can lead to the initiation of indeterminate interpretations even when the biology is benign (9). Cystic change is one practical example of why indeterminate interpretations may be “triggered” by pre-analytical and morphologic confounders: hypocellularity, macrophage-rich backgrounds, degenerative atypia, and limited representation of the lesional follicular epithelium can inflate the AUS rate and complicate downstream management. Clear morphologic criteria and standardized adequacy thresholds remain important in cystic nodules, and they constitute the boundary conditions within which any AI model must operate (10). These realities foster interest in tools that standardize and quantify cytologic interpretation. The persistence of indeterminate categories despite guideline refinements has been characterized as a diagnostic “minefield,” wherein small shifts in thresholds, local prevalence, and cytomorphologic interpretation translate into large differences in surgery rates and patient burden. In this setting, AI is increasingly framed not as a replacement for cytopathologists, but rather a reproducibility and triage layer-standardizing the assessment of borderline patterns, quantifying risk in a calibrated manner, and supporting consistent escalation (repeat FNA, surveillance, molecular testing, or surgery) across institutions (11).

Given the persistent diagnostic uncertainty associated with indeterminate thyroid cytology, there remains a substantial unmet clinical need for tools capable of improving diagnostic reproducibility, reducing unnecessary surgery, and supporting personalized risk stratification. Although advances in cytopathology, molecular diagnostics, and imaging have significantly improved thyroid nodule management, a considerable proportion of patients continue to fall within diagnostic “gray zones” where clinical decision-making remains challenging. Artificial intelligence (AI) has recently emerged as a promising adjunct across multiple stages of the thyroid nodule diagnostic pathway, extending from ultrasound-based risk assessment to digital cytology, computational pathology, and multimodal decision-support systems.

The rationale for this review stems from the rapid expansion of AI applications in thyroid pathology and the growing interest in integrating cytomorphologic, molecular, radiologic, and clinical data into unified predictive frameworks. The objective of this narrative review is to summarize the current evidence regarding AI-assisted approaches in the management of indeterminate thyroid nodules, discuss their potential integration with molecular testing and digital cytology, analyze current limitations and implementation challenges, and highlight future directions for research and clinical translation.

Beyond these commonly recognized pitfalls, indeterminate thyroid cytology encompasses a broad spectrum of challenging morphologic scenarios that collectively contribute to diagnostic uncertainty and variability in clinical management. One of the most frequent challenges is the distinction between hyperplastic nodules and follicular neoplasms, particularly when aspirates display microfollicular architecture with limited colloid. Cytology also remains inherently unable to assess capsular and vascular invasion, precluding definitive differentiation between follicular adenoma and follicular carcinoma and thereby representing a fundamental biological limitation rather than a purely interpretative one. Additional complexity has emerged following the introduction of non-invasive follicular thyroid neoplasm with papillary-like nuclear features (NIFTP), which has altered the traditional relationship between cytologic findings, malignancy risk, and surgical decision-making. Considerable overlap exists among follicular adenoma, NIFTP, and encapsulated follicular variant papillary thyroid carcinoma (FVPTC), all of which may display partially overlapping cytomorphologic features (12, 13).

The interpretation of papillary-type nuclear changes represents another major source of variability. Incomplete, focal, or borderline nuclear atypia often generates atypia of undetermined significance (AUS/FLUS) diagnoses, particularly when nuclear grooves, chromatin clearing, or irregular nuclear contours are present only in a subset of cells or are obscured by technical factors. Similar difficulties arise in Hürthle-cell–predominant lesions, which encompass a biologically heterogeneous spectrum ranging from hyperplastic nodules and Hashimoto thyroiditis to oncocytic adenomas, oncocytic carcinomas, and oncocytic variants of papillary thyroid carcinoma (14). Reactive atypia associated with chronic lymphocytic thyroiditis, degenerative and reparative changes, cystic degeneration, and paucicellular specimens may further obscure interpretation and contribute to indeterminate classifications despite benign underlying biology (9).

Additional diagnostic challenges include microfollicular-pattern lesions with overlapping benign and malignant features, macrofollicular variants that may mimic benign hyperplastic nodules, and the substantial morphologic overlap observed between RAS-like neoplasms and benign follicular proliferations. Rare but clinically important pitfalls include poorly differentiated thyroid carcinoma displaying only subtle cytologic abnormalities, medullary thyroid carcinoma presenting with follicular-like architecture, primary thyroid lymphoma mimicking chronic lymphocytic thyroiditis, and metastatic tumors involving the thyroid gland with misleading cytomorphologic appearances. Furthermore, intranodular heterogeneity and sampling bias may result in incomplete representation of biologically relevant tumor components, limiting the predictive value of cytologic evaluation even when performed by experienced operators (15).

Importantly, many of these challenges are characterized by subjective interpretative thresholds rather than absolute diagnostic criteria. Significant interobserver variability has been documented in the assessment of nuclear grooves, pseudoinclusions, chromatin clearing, oncocytic change, and mild nuclear atypia, contributing to variability in Bethesda category assignment and reported risk of malignancy among institutions (15). Moreover, cytomorphologic appearances do not always correlate directly with underlying molecular alterations, highlighting the biological complexity of follicular-patterned thyroid lesions and reinforcing the need for integrated diagnostic approaches. These limitations provide a strong rationale for the development of AI-assisted tools capable of objectively quantifying morphologic features, improving reproducibility, and supporting risk stratification in diagnostically challenging cases.

## 2 Indeterminate nodules across different classification systems

International practice is dominated by Bethesda, although national systems, such as the Italian system, also attempt to enhance the differentiation of indeterminate categories, which are generally stratified for “low-risk” and “high-risk” indeterminate nodules (16). This is pertinent because the natural history of low-risk indeterminate lesions suggests that there is compatibility between surveillance and the avoidance of immediate surgery (17). Current management guidelines are increasingly stressing the fact that the indeterminate process is no longer viewed as a discrete nodule but as a process of refinement of risk, which includes ultrasound, cytology, repeat biopsies, and long-term behavior (18, 19).

Pediatric indeterminate nodules are a special kind of nodule. The prevalence of malignancies, cut-offs for surgery, and future impacts are different for this age group from their adult counterparts. A recent systematic review has called for standardized pediatric risk stratification and comprehensive models that incorporate more than one domain, wherein artificial intelligence (AI) may potentially contribute if validated for pediatric subjects (20).

## 3 Molecular testing: strengths, limitations, and why AI is now converging with omics

The indeterminate region of molecular diagnostics over the last decade of Bethesda III-IV nodules was significantly impacted by enhancements of “rule-out” confidence, allowing surveillance rather than surgical interventions (21–23). The classic dataset arguing for genetics in indeterminate cytology was refined using meta-analytic analysis, conveying meaningful utility while pointing out differences among platforms (24). More recently, with widespread adoption of genomic classifiers such as NGS-based panels, there have been improvements in analytic performance and success, including research into ThyroSeq v3 genomic classifier performance (25).

Although molecular testing has substantially improved the management of indeterminate thyroid nodules, several limitations continue to restrict its universal clinical applicability. In addition to challenges related to positive predictive value, local calibration requirements, pre-test disease prevalence, and interpretation of isolated low-risk or intermediate-risk alterations (26, 27), important practical barriers remain. These include the relatively high costs of testing, limited availability of specialized molecular pathology laboratories, restricted access in resource-limited healthcare systems, and the lack of complete standardization across testing platforms and molecular classification strategies. Furthermore, the biological and clinical significance of several molecular alterations remains incompletely understood and continues to evolve as additional evidence accumulates. Certain molecular findings may indicate increased neoplastic potential without necessarily predicting clinically significant malignancy, creating uncertainty in patient counseling and management decisions. Consequently, molecular testing should currently be considered one component of a broader risk-stratification framework rather than a definitive standalone diagnostic solution (28). Future progress will likely depend on integrating molecular information with cytomorphologic, radiologic, and clinical variables through multimodal decision-support systems capable of contextualizing molecular findings within the broader biological landscape of each individual nodule.

A significant step forward would be to enable molecular profiling on cytologic samples on a large scale and integrate these results into risk assessment in a way that takes into account molecular features as well as cytologic patterns, risk assessed by ultrasound, and institutional prevalence rates, with AI ‘translating,’ or modeling, these relationships between molecular profiles and cytologic patterns on a large scale, and thereby identifying patients with highest risk and lowest risk for follow-up on cytology. Recent Italian multi-institutional data support the feasibility and clinical relevance of molecular profiling on thyroid cytology specimens (29). However, the incorporation of molecular data into AI-based multimodal risk models remains an emerging step and should not be considered fully validated for routine clinical decision-making.

## 4 Where AI enters the thyroid nodule pathway

AI applications in thyroidology have expanded rapidly into the domains of ultrasound triage, cytology/whole-slide imaging (WSI) interpretation, integrated diagnostic models, and automation of workflows. Radiology literature has actively considered the extent to which AI-assisted ultrasound can match or compete with FNA-based strategies in selected situations and how AI might help reduce unnecessary biopsies (30–33). Guideline frameworks for multiparametric ultrasound evaluation emphasize standardized risk assessment strategies and provide a foundation on which AI systems can be integrated to reduce subjectivity and improve consistency (34).

**Figure 1:**
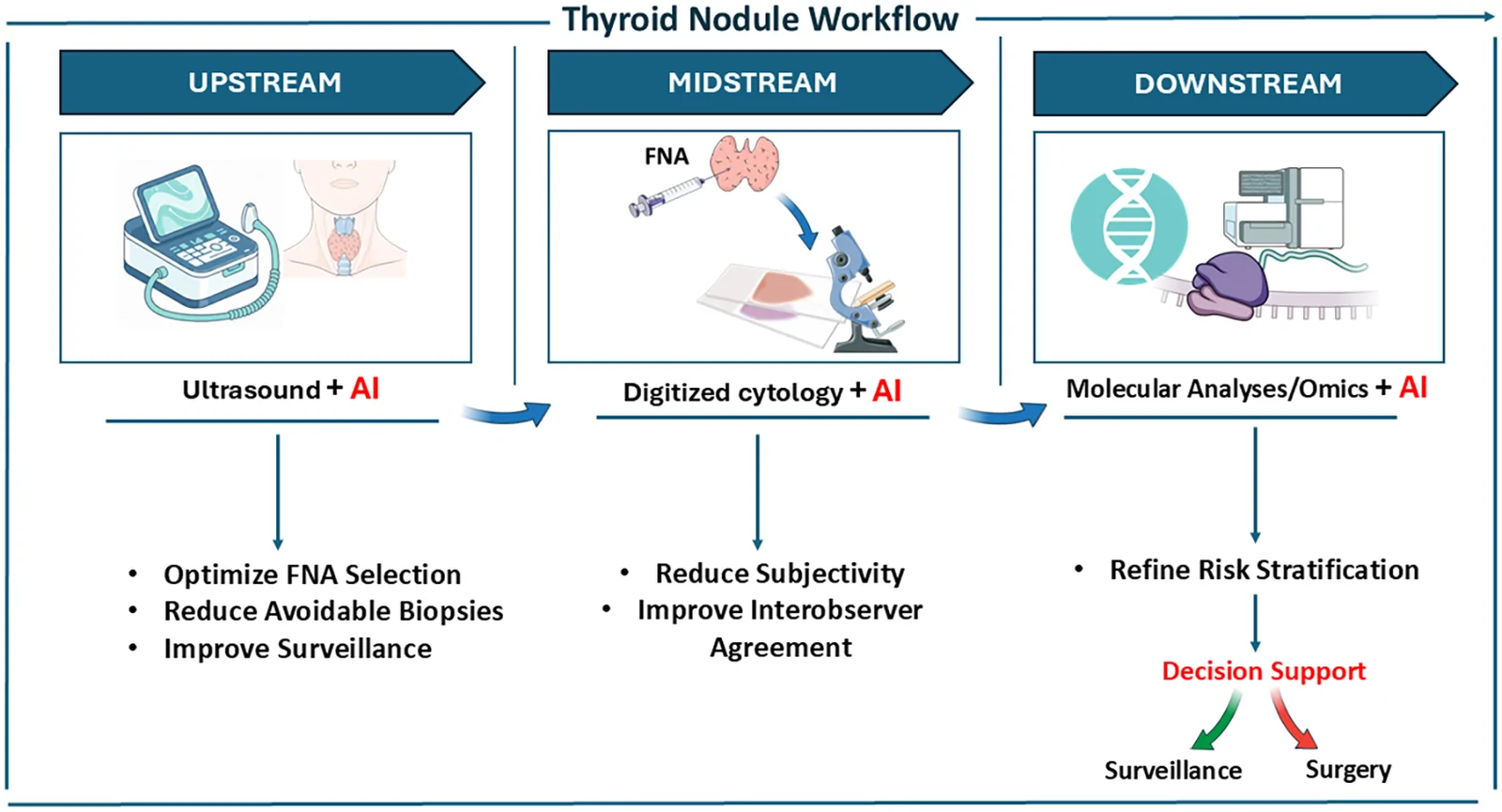
This figure schematically illustrates how artificial intelligence can be incorporated at multiple stages of the thyroid nodule diagnostic workflow, serving as a valuable adjunct to support clinical decision-making. AI, Artificial Intelligence; FNA, Fine-Needle Aspiration (35, 36).

From the workflow perspective, the most realistic near-term use of AI is as a decision-support layer-not as a substitute for clinicians (**Figure 1**):

- upstream: Support a reduction in avoidable biopsies and indeterminate FNAs through improved selection using ultrasound;
- midstream: to decrease interobserver variability in the interpretation of indeterminate cytology;
- downstream: combine cytology with molecular results and imaging to refine post-test probability and choose surveillance vs surgery.

## 5 AI in thyroid cytology: whole-slide imaging and computational pathology

Compared with histology, thyroid FNA cytology poses specific challenges for digitization and AI analysis. Cytologic preparations often contain scattered or overlapping cell groups, variable cellularity, blood or colloid-rich backgrounds, and three-dimensional clusters requiring optimal focusing. In addition, differences between conventional smears and liquid-based preparations, staining protocols, scanner platforms, and z-stack strategies may substantially affect image quality and model generalizability. These pre-analytical and technical variables are not minor implementation details, but central determinants of whether an AI model trained in one institution can be safely transferred to another. Nevertheless, WSI-based cytology offers relevant opportunities, including remote review, telecytology, rapid on-site evaluation support, region-of-interest detection, adequacy assessment, and second-reader assistance in diagnostically challenging or indeterminate cases.

**Figure 2:**
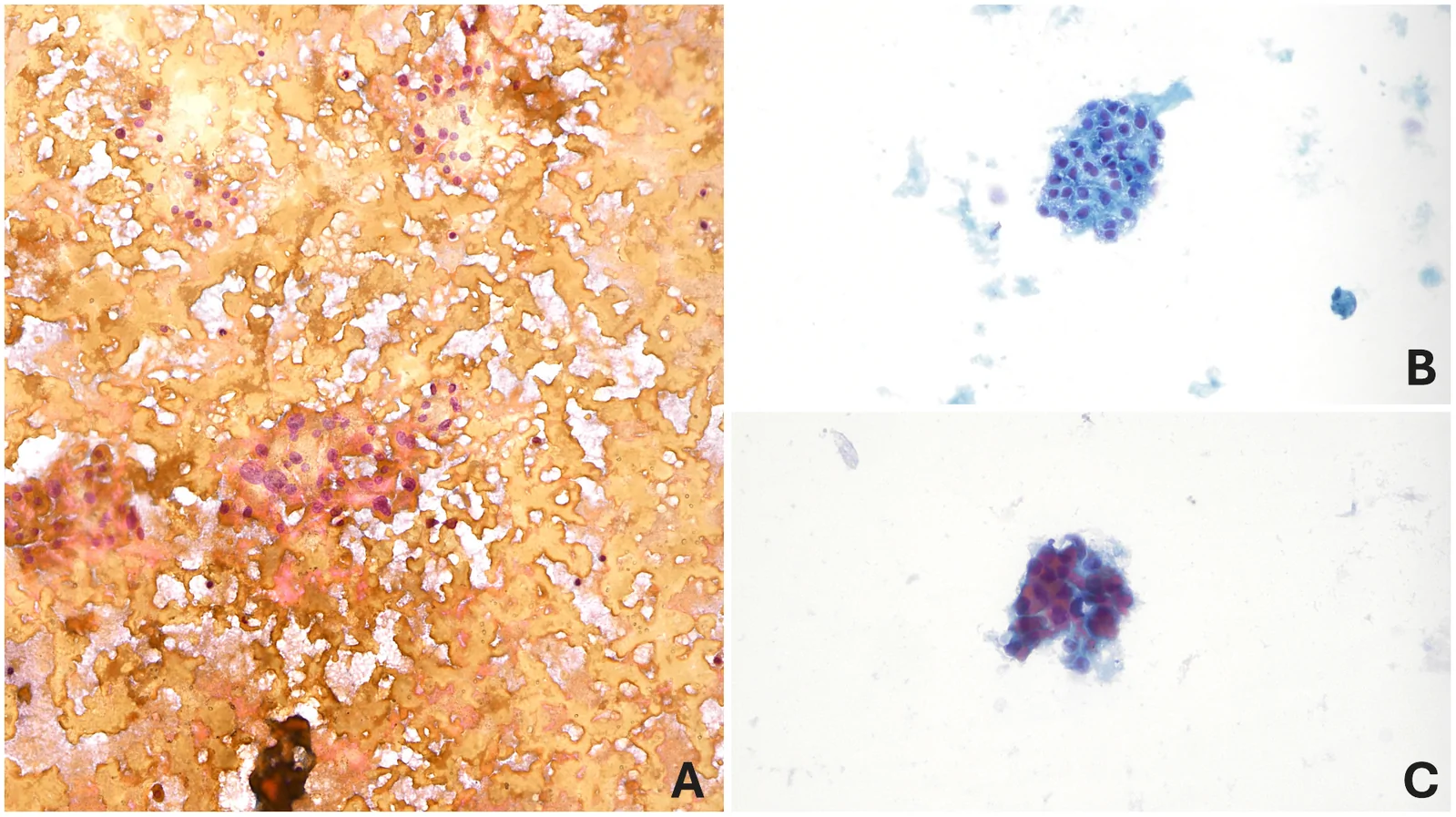
Cytomorphologic challenges in indeterminate thyroid cytology. Representative thyroid fine-needle aspiration cytology images illustrating morphologic patterns that may contribute to indeterminate interpretation. **(A)** AUS case, showing a mixed population of small- and medium-sized follicular cells, the latter with focal nuclear pleomorphism; May–Grünwald–Giemsa stain, 400×. **(B)** FN/SFN case, showing a cohesive follicular epithelial group with nuclear crowding and irregularities; ThinPrep, 200×. **(C)** FN/SFN case, showing a compact oncocytic follicular cell group; ThinPrep, 400×. These examples illustrate morphologic patterns underlying indeterminate thyroid cytology and support the rationale for AI-assisted WSI-based standardization. AI, artificial intelligence; FNA, fine-needle aspiration; WSI, whole-slide imaging.

The cytomorphologic complexity of indeterminate thyroid cytology is illustrated in **Figure 2**. Representative examples include low cellularity, overlapping epithelial groups, oncocytic or degenerative changes, cystic background, and subtle nuclear atypia. These patterns represent precisely the type of borderline morphologic information that may contribute to interobserver variability and that AI-assisted WSI tools should be trained to recognize, quantify, and contextualize.

Recent developments in computational pathology have expanded AI applications beyond simple benign-versus-malignant classification toward automated characterization of specific cytomorphologic patterns. Deep learning algorithms are increasingly being trained to recognize microfollicular architecture, oncocytic change, nuclear atypia, colloid-rich backgrounds, cellularity patterns, and architectural arrangements that contribute to indeterminate diagnoses. Such approaches may improve reproducibility in the assessment of AUS and FN/SFN categories while providing quantitative support for morphologic interpretation (36, 37).

Particular interest has emerged regarding AI-assisted identification of lesions belonging to the RAS-like spectrum, including NIFTP and encapsulated follicular-patterned neoplasms. Because NIFTP occupies an intermediate biological position between benign proliferations and conventional papillary thyroid carcinoma, its recognition remains one of the most challenging areas in thyroid cytopathology. AI-based systems capable of integrating subtle nuclear and architectural features may contribute to more consistent identification of cases likely to belong to this category and reduce interobserver variability (12, 13).

Additional applications include automated slide-screening systems designed to identify suspicious regions requiring pathologist review, adequacy assessment, quality-control monitoring, and prioritization of high-risk cases. Such tools may reduce workload, improve standardization, and facilitate large-scale implementation of digital cytology workflows. Similar AI-assisted workflows have already demonstrated promising results in other areas of cytopathology, supporting adequacy assessment, region-of-interest detection, and second-reader strategies (38, 39).

Importantly, AI-based quality-control systems may also identify technical artifacts, inadequate cellularity, staining variability, focusing defects, and scanning errors before diagnostic interpretation, thereby improving both efficiency and reliability of digital pathology workflows. However, successful implementation requires robust quality assurance procedures encompassing image acquisition consistency, focus verification, slide completeness assessment, scanner calibration, stain normalization strategies, and continuous performance monitoring (40, 41). Such quality-control frameworks are likely to become increasingly important as digital cytology moves from proof-of-concept studies toward routine clinical practice.

This development in WSI has made it possible to algorithmically evaluate cytology slides, especially in the context of indeterminate lesions, in which sensitive pattern recognition and quantification can potentially lessen the subjective nature of AUS classifications. A systematic review concentrating on AI + WSI in the assessment of indeterminate thyroid cytology identified potential utility in using AI to improve the subjective assessment of indeterminate cytology but noted difficulties in comparability and external validation (37) (**Table 1**).

**Table 1:** Overview of the main artificial intelligence tools and models applied to the diagnosis of indeterminate thyroid nodules, reporting sensitivity, specificity, and key outcomes.

Author, Year, Reference	AI Approach	Sensitivity	Specificity	Main Findings
Poursina et al., 2025, (37)	CNN/ANN + WSI	High	Low to High (14.4 % to 100 %)	Improved diagnostic accuracy and reduced ambiguity in indeterminate thyroid nodules
Negrelli et al., 2025, (38)	Deep learning	N/A	N/A	Reduced inter-observer variability and increased standardization
Dov D et al., 2023 (44)	Deep learning approach based on two CNNs	N/A	N/A	The algorithm automatically classified 45.1% of WSIs as benign or malignant, with corresponding risks of malignancy (ROM) of 2.7% and 94.7%, respectively. In addition, 21.3% of indeterminate cases were reclassified as benign with a final ROM of 1.8%. The model achieved diagnostic performances comparable to expert cytopathologists, supporting its potential role in thyroid cytopathology screening and ancillary testing
Dov D et al., 2019 (46)	Two-stage CNN-based deep learning pipeline for WSI thyroid FNAB	N/A	N/A	The model demonstrated diagnostic accuracy comparable to experienced cytopathologists for predicting thyroid malignancy. The study showed that AI-based WSI analysis could support automated screening and improve evaluation of indeterminate thyroid cytology cases by identifying informative cellular regions and assisting malignancy risk stratification
Athreya S et al., 2025, (47)	Multimodal deep learning (US + MT)	94.6 %	66.4 %	Significant improvement in PPV and specificity over molecular testing alone
Cai X et al., 2025, (48)	Five different AI models (AI-assisted US + cytology + clinical data)	89.5 %	91.3 %	RF model showed superior overall performance and robust prediction capability. Accuracy of 90.5% for indeterminate thyroid nodules (Bethesda III–IV)
Sun et al., 2022, (50)	ANN combined with high-throughput proteomics. The model used a panel of 19 protein biomarkers selected via genetic algorithms and neural network optimization to classify thyroid nodules as benign or malignant from FFPE and FNA samples	84% (retrospective FFPE cohort); 85% for FNAB	94% (retrospective FFPE cohort); 70% for FNAB	The AI-based proteomic classifier achieved AUC values of 0.93–0.94 and overall accuracies of 89% in retrospective FFPE cohorts and 85% in prospective FNA cohorts. For indeterminate Bethesda III/IV nodules, the model achieved an AUC of 0.89, demonstrating strong potential as an adjunctive diagnostic tool for improving thyroid nodule classification and reducing unnecessary surgeries
Carnabatu CJ et al., 2025, (55)	AI-based decision support software (Koios DS)	85.7 %	76.2 %	AI improved PPV from 33.3 % to 54.5 % while maintaining high NPV (94.1 %), reduced available biopsies, supported more precise decision-making in indeterminate cases, and provided TI-RADS assessments aligned with radiologists

CNN, Convolutional Neural Networks; ANN, Artificial Neural Networks; WSI, Whole Slide Imaging; US, Ultrasound; MT, Molecular Testing; AI, Artificial Intelligence; RF, Random Forest; PPV, Positive Predictive Value; NPV, Negative Predictive Value; NA, Not Available; TI-RADS, Thyroid Imaging Reporting and Data System; FNA, FNAB, Fine Needle Aspiration Biopsy; AUC, Area Under the Curve.

The reviews focused on cytopathology highlight technical challenges which play even more important roles in the field of cytology such as stain variation, sample variation (smear and liquid-based cytopathology), scanner variation, and domain transfer between institutions because they may harm CNN model performance in settings different from those it was trained in (41). These challenges are consistent with the view found in the area of pathology which holds computationally integrated cytopathology as the probable future, but only when standardized on an appropriate level (42).

A variety of explanatory techniques can be employed with various deep learning architectures, including CNN, autoencoder, or recurrent neural networks. Although not thyroid-specific, the work of Marya et al. (43) in other cytology domains has shown how AI-assisted applications can support adequacy assessment, ROI detection, and second-reader workflows. For indeterminate classes, the focus on "explainability" has to be actionably informed, with clear indications of which cell groups/fields are most important drivers of classification with outputs calibrated against local malignancy risk rather than thresholds, where the latter will often be a key factor rather than continuous improvement in AUC.

Again, in all clinically oriented research in WSI with a high scientific stringency level, “assistive deployment” is a constant theme in emphasizing the role of AI in improving reproducibility and efficiency beyond human judgment in cytopathology. Furthermore, in “image gallery” type of implementation studies in ROI-screening for WSI, it is observed as having almost perfect correlation in WSIS in comparison to traditional WSI in a series of thyroid FNA samples. A high level of research implementation, with multicenter model testing in terms of excluding bias in testing WSI implementation, is described as being highly discriminatory in “Bethesda III+” type WSI, together with notable “reader-impact” in terms of improving specificity as well as accuracy in terms of improving the quality of judgments in WSI, specifically in younger cytopathologists assisted by WSI. Complementary approaches include “ancillary tests” in terms of “confident” classification of certain samples as being in very low risk or high risk in greatly reducing the “gray zone” while maintaining safety in WSI samples (44–51).

## 6 Multimodal AI: integrating ultrasound, cytology, and molecular features

The strongest conceptual argument for AI in indeterminate thyroid nodules is that no single modality captures the entire diagnostic and biological signal. Ultrasound, cytology, molecular testing, clinical variables, and local ROM each provide partial information. Multimodal AI aims to combine these data streams to refine post-test probability, ideally improving PPV while preserving a sufficiently high NPV to support safe surveillance in selected patients. However, most available evidence remains retrospective or early-stage, and the clinical utility of multimodal AI still requires prospective validation, standardized input data, and clearly defined decision thresholds.

Athreya et al. demonstrated the potential value of combining ultrasound imaging and molecular testing in a multimodal deep learning model for risk stratification of indeterminate thyroid nodules (47). Cai et al. further showed that models integrating AI-assisted ultrasound, cytology classification, and clinical data may improve diagnostic performance in Bethesda III–IV nodules (48). Other machine-learning approaches incorporating immunologic, radiologic, cytologic, and radiomic features also support the principle that structured feature fusion may outperform isolated input streams (49). Together, these studies suggest that integration, rather than replacement of established diagnostic modalities, is likely to represent the most clinically realistic role of AI in this field. Other related modeling efforts that integrated immunologic, radiologic, cytologic, and radiomic features also directed toward the same routes: integration beats isolation for many indeterminate pathways (50). A practical "what to do next" plan which might have a high probability of moving us closer to a practical answer involves a series of steps: ultrasound "risk stratification" to optimize those biopsies as a first cut through the problem; digital cytology and ROI screening navigation as a second step to reduce the burden on human observers and increase standardization; "Bethesda-aligned" AI output to potentially decrease the number of indeterminate results; and as a final option, feature fusion to increase the positive predictive probability, keeping the high negative predictive probability safe but improving surveillance outcomes.

The aim is not to supersede existing guidelines, but to support their consistent and risk-adapted application across different clinical settings, with the goal of reducing unnecessary procedures while maintaining oncologic safety.

As an example, a thyroid nodule detected by US shows a solid architecture and is mildly hypoechoic, wider-than-tall, with smooth margins and no microcalcifications, corresponding to an intermediate-risk category on conventional ultrasound risk stratification. Fine-needle aspiration (FNA) is performed, and cytology is reported as Bethesda III (AUS) due to mild nuclear atypia in a background of low cellularity.

At this stage, traditional management options would include repeat FNA, molecular testing, or diagnostic lobectomy, depending on institutional practice and patient preference. In an AI-assisted workflow, the case is instead processed through a multimodal decision-support system integrating three data streams:

1. Ultrasound AI module: a deep learning algorithm analyzes stored ultrasound images and assigns a malignancy probability of 8%, reclassifying the nodule as low risk within its internal model despite intermediate conventional ultrasound features.
2. Cytology AI module (WSI-based): digitized cytology slides are analyzed by a convolutional neural network trained on indeterminate thyroid nodules. The system quantifies nuclear irregularity, crowding, and chromatin texture, classifying the sample as “AUS—low-risk pattern,” with features overlapping benign hyperplastic nodules rather than follicular neoplasia.
3. Molecular–AI integration layer: Targeted molecular testing reveals an isolated RAS mutation without additional high-risk alterations. Rather than interpreting this result as an automatic indication for aggressive surgery, the AI model integrates the molecular finding with low-risk ultrasound and cytologic features, recalibrating the overall risk profile and supporting an individualized discussion between surveillance and diagnostic lobectomy.

The final output is presented to the clinician as an interpretable decision-support report, indicating: (i) low integrated malignancy risk, (ii) absence of features associated with clinically aggressive disease, and (iii) recommendation for ultrasound surveillance rather than immediate surgery. The cytopathologist remains responsible for the final diagnosis, while the AI system functions as a quantitative second reader and risk integrator.

This example illustrates how AI does not replace cytologic expertise or molecular testing, but may help contextualize borderline or discordant findings, reduce overinterpretation of isolated molecular alterations, and support shared decision-making. Importantly, the value of AI in this context lies not in binary classification alone, but in continuous risk refinement aligned with clinically meaningful endpoints, such as avoidance of unnecessary surgery, safe surveillance, and appropriate escalation of care when risk features accumulate.

## 7 Beyond genomics: proteomics, spatial profiling, and AI-defined protein signatures

The diagnostic frontier is extending beyond DNA/RNA alterations toward proteomics, spatial profiling, and AI-defined protein signatures. AI-driven proteomic classifiers have shown that protein-expression profiles can contribute to the distinction between benign and malignant thyroid nodules (50). In parallel, spatial proteomics studies on indeterminate thyroid nodules have identified candidate biomarkers that may further refine lesion classification and support the development of multi-level diagnostic ecosystems (51). Alongside these “omics-heavy” approaches, immunocytochemistry remains one of the most accessible ancillary methods in routine pathology laboratories. For example, PD-L1 assessment on thyroid cytologic samples has been explored in oncocytic thyroid lesions and oncocytic change, suggesting that digital quantification and AI-assisted image analysis could reduce interobserver variability in selected settings (52). Similarly, immunocytochemical biomarkers in FNAC samples may supplement the distinction between benign and malignant thyroid lesions, especially where molecular diagnostics are not available (53).

However, concurrently, immunocytochemistry has continued to play its part in lymph node assessment, particularly in settings where molecular diagnostics are not available. A meta-analysis of immunocytochemical biomarkers in FNAC has identified important markers that can supplement diagnosis to distinguish between benign and malignant lesions, and this can, in turn, be improved through digital image analysis and AI algorithms (54).

## 8 Clinical meaning: focusing on outcomes that matter

The true clinical goal is not merely to improve AUC, but to reduce avoidable biopsies, limit unnecessary diagnostic surgery, optimize the use of molecular testing, and preserve the safety of surveillance strategies for low-risk disease. The validation of AI decision support tools in indeterminate nodules has shown promising performance of these tools in reducing biopsies in low-risk patients while upholding negative predictive value (NPV), a highly desirable outcome from a health infrastructure perspective (55). Demonstrating clinical utility will also require implementation outcomes beyond discrimination metrics: cost-effectiveness analyses, such as those detailing the number of diagnostic surgeries avoided or optimized molecular testing use; workflow measures, including turnaround time and need for repeat procedures; and prospective monitoring of error modes in specific morphologic subtypes, such as Hürthle-cell-predominant aspirates. AI systems may also have an educational role in supporting trainees as a "second reader" that highlights overlooked nuclear or architectural cues, provided that safeguards against automation bias are incorporated and performance is continuously audited in local practice.

On the other hand, the residual malignancy risk among cytologically low-risk indeterminate nodules managed by surveillance nodules makes a future role for AI in standardizing the follow-up intensity and identifying which nodules should be escalated to repeat FNA, molecular testing, closer follow-up, or surgery (17, 31). This is reflected in the larger management guidelines and controversy regarding indeterminate nodule management, where the balance of overtreatment and undershoot of clinically significant malignancies is the challenge (18, 19).

## 9 Current limitations and requirements for clinical translation

Across systematic reviews and technical papers, several constraints consistently limit real-world deployment:

1. pre-analytic variability (smears vs LBC, fixation, stain);
2. scanner and digitization heterogeneity;
3. domain shift across institutions;
4. selection bias from “surgery-only ground truth” cohorts;
5. interpretability and workflow integration requirements;
6. quality-control requirements for digital cytology workflows, including image acquisition consistency, focus verification, slide completeness assessment, and continuous performance monitoring (37, 38, 55).

Translated, four features appear throughout the most clinically mature literature: i) external validation across preparations-smears vs LBC, stains, scanners, and institutions; ii) calibrated probabilities and clinically justified thresholds reflecting local ROM; iii) prospective evaluation with readerimpact analyses, predefined workflow endpoints that include reduced benign surgery, safe surveillance, and optimized molecular testing; and iv) QA of digitization as a precondition rather than afterthought, including focus metrics, scan failure policies, and explicit z-stack strategies. Finally, post-deployment monitoring is essential: keeping a log of AI suggestions and the clinical outcomes allows detecting systematic failure modes and enables a controlled pathway to model updates ("adaptive AI under oversight"), while keeping traceability and patient safety.

A systematic review and meta-analysis focusing on AI-powered diagnostics for indeterminate nodules concluded that performance can be strong in controlled conditions, but generalizability and clinical readiness depend on rigorous external validation and standardized methodologies (55). Practically speaking, good AI in thyroid cytopathology needs to be appropriate to local ROM, understandable to be trusted by clinicians, and validated with respect to meaningful endpoints, such as decreasing the number of procedures, safety, and inter-rater consistency (55).

## Conclusions

AI is increasingly being viewed not as a replacement for cytopathologists or molecular diagnostics, but as a complementary layer capable of improving standardization, reproducibility, and multimodal risk assessment in patients with indeterminate thyroid nodules. Current evidence suggests that the most promising short-term applications include automated adequacy assessment, AI-assisted identification of diagnostically relevant regions on digital cytology slides, reduction of interobserver variability within Bethesda III and Bethesda IV categories, and integration of ultrasound, cytologic, molecular, and clinical data into unified risk-prediction models.

From a practical clinical perspective, AI-assisted approaches may help reduce unnecessary diagnostic surgery, optimize the use of molecular testing, support surveillance strategies in appropriately selected patients, and improve consistency of risk stratification across institutions. However, routine implementation remains limited by variability in slide preparation, digitization protocols, molecular testing availability, and the lack of large externally validated datasets.

Future research priorities should include prospective multicenter validation studies, harmonization of digital cytology workflows, development of explainable AI systems, creation of internationally standardized datasets, and evaluation of clinically meaningful outcomes such as reduction in diagnostic surgery, optimization of molecular testing utilization, cost-effectiveness, and patient quality of life. In addition, further investigation is needed regarding AI-assisted identification of NIFTP and other follicular-patterned lesions, automated quality-control systems, and multimodal platforms capable of integrating molecular, radiologic, cytologic, and clinical information.

Ultimately, successful clinical implementation will depend not only on algorithmic performance but also on transparency, regulatory oversight, interoperability with existing pathology workflows, and continuous quality-control monitoring. If these challenges are adequately addressed, AI may become an integral component of precision triage strategies for indeterminate thyroid cytology, helping clinicians balance diagnostic accuracy, patient safety, and avoidance of unnecessary treatment.
