# Correction: The role of artificial intelligence in thyroid cytology of indeterminate nodules: from digital cytology to multimodal precision triage

**DOI:** 10.3389/fendo.2026.1950315

**Published:** 2026-08-06

**Authors:** Pietro Tralongo, Mariagiovanna Ballato, Vincenzo Fiorentino, Valeria Zuccalà, Cristina Pizzimenti, Ludovica Rita Pepe, Angelica Cardile, Teresa Maria Martorana, Antonio Ieni, Maurizio Martini, Guido Fadda

**Affiliations:** 1 Department of Biomedical, Dental and Morphological and Functional Imaging Sciences, University of Messina, Messina, Italy; 2 Department of Human Pathology of the Adulthood and of the Developing Age “Gaetano Barresi”, University of Messina, Messina, Italy; 3 Pathology Unit, Papardo Hospital, Messina, Italy

**Keywords:** AI-assisted analysis, cytology, indeterminate nodules, thyroid, whole slide analysis

The funder “University of Messina, APC Initiative to Dr. Pietro Tralongo” was erroneously omitted. The **Funding** statement has been corrected to read:

“The author(s) declared that financial support was received for this work and/or its publication. University of Messina, APC Initiative to Dr. Pietro Tralongo.”

The original version of this article has been updated.

